# Effect of IPTp on *Plasmodium falciparum* antibody levels among pregnant women and their babies in a sub-urban coastal area in Ghana

**DOI:** 10.1186/s12936-017-1857-1

**Published:** 2017-05-26

**Authors:** Judith K. Stephens, Eric Kyei-Baafour, Emmanuel K. Dickson, Jones K. Ofori, Michael F. Ofori, Mark L. Wilson, Isabella A. Quakyi, Bartholomew D. Akanmori

**Affiliations:** 1 Biological, Environmental and Occupational and Health Sciences Department, School of Public Health, College of Health Sciences, University of Ghana, P. O. Box LG 13, Legon, Accra, Ghana; 2 Immunology Department Noguchi Memorial Institute for Medical Research, College of Health Sciences, University of Ghana, P. O. Box LG 581, Legon, Accra, Ghana; 3 Vaccine Research and Development, Immunization and Vaccines Development Cluster, Office of the Regional Director, WHO Regional Office for Africa, P. O. Box 06, Djoue, Brazzaville Congo; 4 Department of Epidemiology, School of Public Health, The University of Michigan, 109 Observatory Street, Ann Arbor, MI 48109-2029 USA

**Keywords:** Malaria, Pregnancy, Antibodies, IPTp, Placenta, Cord blood, Sulfadoxine–pyrimethamine

## Abstract

**Background:**

Women exposed to *Plasmodium* infection develop antibodies and become semi-immune. This immunity is suppressed during pregnancy making both the pregnant woman and the foetus vulnerable to the adverse effects of malaria, particularly by *Plasmodium falciparum.* Intermittent preventive treatment of malaria in pregnancy (IPTp) with Sulfadoxine–pyrimethamine (SP) tablets is one of the current interventions to mitigate the effects of malaria on both the pregnant woman and the unborn child. The extent to which IPTp may interfere with the acquisition of protective immunity against pregnancy-associated malaria (PAM) is undefined in Ghana.

**Methods:**

Three-hundred-and-twenty pregnant women were randomly enrolled at the antenatal clinic (ANC) in Madina, Accra. Venous blood samples were obtained at first ANC registration and at 4-week intervals (post-IPTp administration). Placental and cord blood samples were obtained at delivery and the infants were followed monthly for 6 months after birth. Anti-IgG and IgM antibodies against a crude antigen preparation and the glutamate-rich protein (GLURP) of *P. falciparum* were quantified by the enzyme-linked immunosorbent assay (ELISA).

**Results:**

There was a general decline in the trend of mean concentrations of all the antibodies from enrolment to delivery. The levels of antibodies in cord blood and placenta were well correlated. Children did not show clinical signs of malaria at 6 months after birth.

**Conclusions:**

IgG against both crude antigen and GLURP were present in placenta and cord blood and it is therefore concluded that there is a trend of declining antibody from enrolment to delivery and IPTp-SP may have reduced malaria exposure, however, this does not impact on the transfer of antibodies to the foetus in utero. The levels of maternal and cord blood antibodies at delivery showed no adverse implications on malaria among the children at 6 months. However, the quantum and quality of the antibody transferred needs further investigation to ensure that the infants are protected from severe episodes of malaria.

## Background

About 50 million women who become pregnant in malaria-endemic regions each year [1] are infected with *Plasmodium* parasite [2]. Pregnant women are three times more likely to suffer from severe disease from malarial infection than their non-pregnant counterparts, with close to 50% mortality rate from severe disease [3]. Malaria, particularly *Plasmodium falciparum* infection increases the risk of maternal anaemia and other complications in pregnancy, often leading to abortions and stillbirths [4].

In general, immunity to *P. falciparum* develops slowly and naturally [5, 6] after repeated exposure [7–10]. The acquired anti-*Plasmodium* antibodies confer protective immunity [11], which reduces morbidity [12–14]. However, clinical immunity to malaria is partial and short-lived, and requires constant ‘longitudinal exposure’ to parasites to be maintained [9].

Despite acquiring partial immunity after years of exposure to *Plasmodium*, pregnant women become highly susceptible to *P. falciparum* due to the absence of immunity to pregnancy-specific isolates that sequester in the placenta [2, 15] resulting in long-standing placental malaria even when peripheral blood may be negative for malaria parasites, especially during the second trimester [16].

The parasites that infect the placenta have a special affinity to accumulate in the erythrocytes in the blood spaces of the placenta [17] and also characteristically bind to the placental ligand, chondroitin sulfate A (CSA) [18, 19]. A protective anti-CSA antibody-mediated immunity to pregnancy-associated malaria (PAM) develops after repeated exposure of the placenta to parasites. The multigravidae, who have developed anti-CSA from previous exposure, are less susceptible to PAM than the primigravidae who are experiencing the placenta for the first time [20, 21]. PAM can affect placenta development and function, leading to deleterious outcome to both mother and her infant [22–24], which further increases the risk for neonatal and infant mortality [25, 26]. Infection of the placenta by malaria parasites has been found to have no effect on the transmission of maternal antibodies to infants [27].

Another reason for the high susceptibility of pregnant women is attributed to various hormonal and immunological changes during pregnancy, with a leaning from cell-mediated immunity to humoral immunity. Primigravidae have higher levels of cortisol than multigravidae and therefore lose their acquired immunity more frequently and this increases their susceptibility to *P. falciparum* infection. These problems are more common in the first and second pregnancies since the parasitaemia level generally decreases with increasing numbers of pregnancies. In Ghana, malaria during pregnancy increases maternal anaemia and low birth weight (LBW), especially in women living in rural communities [28–30].

Newborn infants in endemic areas are markedly resistant to *falciparum* malaria due to protection from high levels of maternal anti-malarial immunoglobulin G (IgG) passively transferred across the placenta [31]. Congenital malaria is therefore rare in endemic areas but infants may present with parasitaemia within 2–3 months as the maternal acquired antibodies wane [32]. There is therefore a gradual acquisition of IgG to variant surface antigens (VSAs) after birth, while protection from maternal VSA-specific IgG steadily declines [33]. These antibodies, against a broad range of variant antigens, are important for protection against febrile malaria episodes in children who are in the process of acquiring malaria immunity [13]. Severe disease is therefore rare during the first few months of life [16].

The use of intermittent preventive treatment of malaria in pregnancy (IPTp) has been shown to increase birth weight and reduce the incidence of LBW [34] as well as reduce pregnancy-related parasitaemia and anaemia [35–40]. Regardless of these benefits, there is concern that “any exposure-reducing interventions could result in the loss of or failure to acquire protective (PAM) immunity” [41–43] which would increase the overall, long-term disease burden [44] by increasing morbidity and mortality [45, 46]. The use of IPTp with Sulfadoxine–pyrimethamine (SP) treatment of primigravidae has also been shown to reduce levels of plasma IgG, which protects against pregnancy-associated *falciparum* malaria [47, 48] and severe malaria among children [49]. However, the extent to which IPTp may interfere with acquisition of protective immunity against PAM is largely undefined, particularly in Ghana. The present study was designed to evaluate the effect of IPTp on antibody-mediated immunity against malaria during pregnancy. This study further sought to determine whether IPTp disrupts the transfer of maternal antibodies across the placenta. Specific concentrations of IgG and immunoglobulin M (IgM) antibodies against a glutamate-rich protein (GLURP) and crude antigen (Ag) preparation were obtained from pregnant women from enrolment to delivery and from cord blood of the infants.

## Methods

### Study setting

The study was conducted at the Alpha Medical Hospital in Madina, a densely populated and fast-growing, peri-urban area in the Ga-East Municipality of Accra, southern Ghana. This study was a sub-component of a larger study that looked at IPTp implementation in the Ga-East Municipality of Accra. The full details of the study setting and characteristics of the participants are described in Stephens et al. [50].

### Study participants

The study participants were selected from all pregnant women making their first visit for antenatal care at the Alpha Medical Centre from July to August regardless of ethnic or socio-economic background, gestation, parity or gravid status. The inclusion criteria were pregnant women who indicated their intention to deliver at the facility and who had not previously taken IPTp for their current pregnancy.

### Study procedure

Briefly, pregnant women who met the inclusion criteria were invited to volunteer to participate in the study. Socio-demographic information was collected by questionnaire, as well as information on gravidity, parity, the use of IPTp, insecticidal-treated nets (ITNs), drug seeking behaviour, and other anti-malarial practices during pregnancy. Gestation was estimated from abdominal examination by qualified midwives and confirmed later by scan. A venous blood sample was obtained at first registration for general screening and for plasma analysis to determine pre-IPTp antibody status. Women who were eligible for IPTp administration were served by hospital staff at monthly intervals. Blood samples were then obtained 4 weeks after each IPTp administration to determine their post-IPTp *P. falciparum* antibody titre. Placenta and cord blood samples were also collected at delivery. Infants born to these women were followed at monthly intervals for 6 months. The body temperature of the infants was determined at each visit. Follow-up was facilitated by the use of mobile phones.

### Blood sampling

Venous blood was collected into 5-ml K_3_EDTA vacutainer tubes with butterfly needles (Becton–Dickinson Vacutainer Systems, UK) by a qualified phlebotomist. Each tube was labelled with the subject’s unique identification number and stored in a cool box. Samples were transferred to the laboratory at the University of Ghana, Legon for further analysis. Whole blood samples were centrifuged at 2000 revolutions per minute (rpm) using a Spectrafuge (Labnet International, Inc, Edison, NJ, USA) and the plasma was carefully collected and stored at −40 °C for anti-malaria antibody assays. The resulting pellet of red cells were washed twice and then cryopreserved in glycerolyte solution at −40 °C.

Maternal intervillous blood (IVB) was obtained at delivery by a modified placental prick method as described by Othoro et al. [51]. An experienced technician accessed the placental intervillous spaces through the chorionic plate after delivery. The placenta was carefully placed on a raised sterile wire mesh stand, with the chorionic plate facing downward to facilitate blood accumulation and IVB space. A large-bore, 14-gauge needle attached to a syringe was directed approximately 0.5 cm deep through the wire mesh into the intervillous space, denoted as dark purple regions, taking care to avoid puncturing the surrounding fetal vessels on the surface of the chorionic plate. The syringe was gently pulled to create a vacuum and initiate blood flow. The needle was withdrawn to collect a 5-ml sample into a previously labelled micro centrifuge tube charged with 25 μl of a 1:4 heparin (stock concentration, 1000 units/ml) dilution in phosphate-buffered saline (PBS). The procedure was repeated to collect cord blood. The samples were stored in a refrigerator at the maternity unit and transferred in a cool box to the laboratory for further processing. Birth weight of each infant was recorded by the delivering midwife.

### Antibody analysis

Antibodies against two antigens, a specific blood-stage antigen of *P. falciparum* or GLURP, and a soluble crude antigen preparation from whole blood-stage parasites were determined as previously described [52]. Briefly, 96-well micro titre plates (Immulon 2 HBX) were coated (100 μl/well) at 1 μg/ml for GLURP and 5 × 10^5^ cells/well for crude antigen in plain PBS. The plates were incubated overnight at 4 °C, retrieved, washed four times with PBS/Tween 20, then incubated with 200 μl/well of blocking buffer (3% skimmed milk in 1X PBS) for 1 h at room temperature in a humidified chamber. The plates were washed as described and 100 ul of plasma pre-diluted at 1:2000 in 1% PBS/milk was added in duplicates to each well and incubated for 2 h as described. To control for inter-assay variations in the procedure, each assay plate included a pool of hyper immune plasma known to be positive for total IgG to GLURP and the schizont extract, diluted at 1:500. To the plates were then added 100 μl/well of 0.5 μg/ml peroxidase labelled goat anti-human IgG (H + L) conjugate (KPL), and peroxidase-labelled goat anti-human IgM at 0.5 μg/ml for 1 h at room temperature for the IgG and IgM respectively and incubated for 1 h. Plates were developed with 100 μl/well 3,3′ 5,5′ tetramethylbenzidine (TMB) substrate for 30 min in the dark. After incubation the reaction was stopped with 100 μl 0.2 M H_2_SO_4_ and optical density (OD) was read at 450 um with a reference wavelength of 630 um using a spectrophotometer (Vmax Kinetic micro plate reader-Molecular Devices Corporation, CA, USA). Optical density values were converted to concentrations using a 4-parameter curve fit using pure human IgG and IgM standards fitted on each plate.

### Ethical considerations

Ethical clearance for the study was obtained from the Institutional Review Board (IRB) of the Noguchi Memorial Institute for Medical Research, College of Health Sciences, University of Ghana, Legon (Study Number: 004/06-07). An informed consent was obtained from each volunteer and assent was documented by signing or by thumb printing the consent form.

### Statistical analysis

Responses to the questionnaire were numerically coded on paper and later transferred into an electronic database. Responses and laboratory results were entered into a computer in Epi Info version 3.5. The data were then cleaned and verified. Data summaries (frequencies and distributions) were examined. Participants were grouped by gravidity and by gestation at registration. The mean values for various parameters, such as antibody levels, use of bed nets and the number of IPTp doses administered for various categories of pregnant women, were compared using standard parametric and non-parametric statistical methods using the SPSS and EPI info statistical software.

For normally distributed data, comparisons between means and percentages were done by Students’ t test, ANOVA and Fisher’s exact tests as appropriate at P < 0.05 significant level. Multivariate regression was performed with the number of IPTp doses taken and antibody titre against age, parity and gestation.

### Limitations

Some limitations are acknowledged. First, HIV status is important in antibody reduction but data were not collected and therefore were not investigated in the present study. Second, it was not possible to examine the association between malaria antibody levels and bed nets due to the low usage of ITNs. Third, although malaria transmission is seasonal with a peak during the rainy season (July to September), the very low prevalence of malaria, reported in an earlier study [50] among the pregnant women, limited the ability to assess seasonality effects.

## Results

**Fig. 1:**
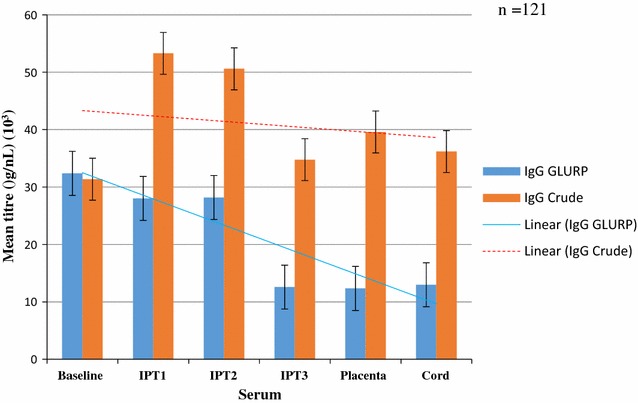
Mean crude and GLURP concentrations of IgG among pregnant women of all parities after IPTp from enrolment to delivery

**Fig. 2:**
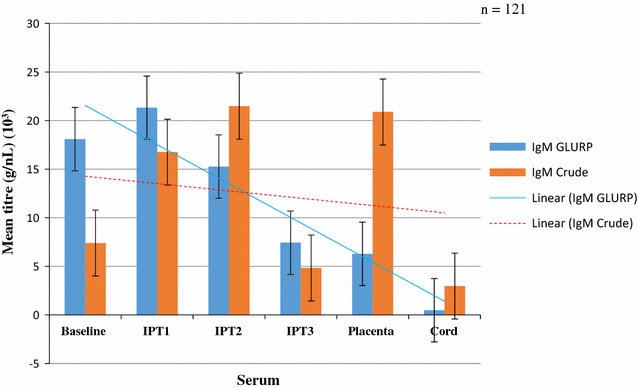
Mean crude and GLURP concentrations of IgM among pregnant women of all parities after IPTp from enrolment to delivery

A total of 320 pregnant women were enrolled in the study. The details of the characteristics of the study participants have been reported in [50]. In brief, the mean gestation at registration and onset of IPTp uptake were 18.5 and 23.5 weeks, respectively. Peripheral blood parasitaemia and placental positivity were 5 and 2.5%, respectively. There were 37.8% (121/320) deliveries in the hospital. One-hundred-and-twenty-one paired placenta and cord blood was obtained at delivery. The rest of the women either did not deliver in the hospital as they had indicated and a few were missed at the time of delivery. The mean concentrations of antibodies against IgG and IgM are shown in Figs. 1 and 2, respectively.

The mean concentrations of IgG against GLURP decreased from the baseline to post IPT1 and slightly increased after IPT2, dropped after IPT3 and plateaued, but imposition of trend lines showed a sharp decrease in trend from baseline to delivery. IgG antibodies against the crude malaria parasite Ag was lower than IgG against GLURP at baseline (pre-IPTp) but increased after the first dose of IPT (IPT1) and then declined until after IPT2 and then decreased at delivery (placental blood) (Fig. 1). The trend lines showed a general decrease. The mean antibody concentrations of IgG against crude Ag were significantly higher than the corresponding concentration of anti-GLURP IgG throughout pregnancy, except at baseline (P < 0.01).

The mean concentrations of anti-GLURP IgM antibodies were higher than those of the anti-crude IgM antibodies at baseline and after IPT1 but the levels reversed after IPT2 and after IPT3 and again at delivery. The levels of IgM antibodies against both Crude Ag increased then decreased after IPT3 but the general trend showed a decrease in both. IgM GLURP also showed a general decrease from post-IPTp1 (Fig. 2). Concentrations of anti-GLURP IgM antibodies were significantly higher than concentration of anti-crude IgM antibodies at baseline but reversed at delivery (P = 0.01).

**Fig. 3:**
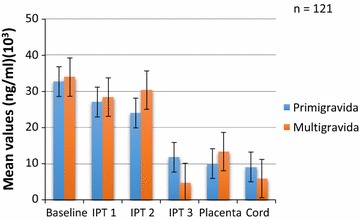
Comparison of IgG GLURP concentration by parity among pregnant women on IPTp

**Fig. 4:**
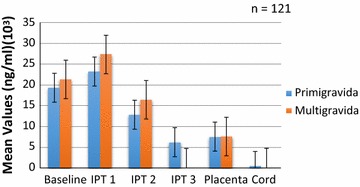
Comparison of IgM GLURP concentration by parity among pregnant women on IPTp

**Fig. 5:**
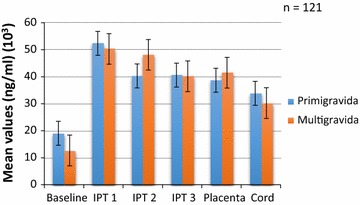
Comparison of crude IgG concentration by parity among pregnant women on IPTp

**Fig. 6:**
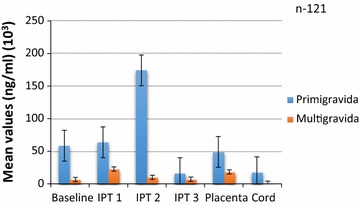
Comparison of crude IgM concentrations by parity among pregnant women on IPTp

The concentrations by parity are shown in Figs. 3, 4, 5 and 6. Analysis of the differences between the mean plasma concentrations of antibodies (IgG and IgM) against the two malaria parasite antigens in primigravidae and multigravidae showed no significant differences. There were significant differences for the mean antibody values of both IgM crude and IgM GLURP (P = 0.01) but no significant differences between IgG crude and IgG GLURP (P = 0.195). Majority 96.7% (117/121) of the babies delivered weighed at least 2.5 kg. Of the few women 3.3% (4/121) who delivered babies with birth weight less than 2.5 kg, two had taken one dose and the other two had taken two doses of IPT. The data showed that one of them was a primigravida, two were multigravidae and the gravid status of the fourth person was unknown. Among those who delivered babies of at least 2.5 kg, 7.7% (9/117) had not received any IPT during pregnancy, while 46.2% (54/117) received the three doses of IPT, which was the maximum number of recommended doses at the time of the study. A significant correlation was found between the number of IPTp doses taken and the weight of the baby at delivery (P = 0.024). There was also a strong positive correlation between antibody levels of placental blood and cord blood.

Anti-GLURP IgG levels declined from baseline to delivery for the primigravida while mean concentrations among the multigravida declined at baseline but the level increased after IPTp2 and then decreased after IPTp3 (Fig. 3). Both anti-GLURP IgG and IgM concentrations for the multigravida were higher than those for the primigavida except at IPTp3 where the levels for the multigravida dropped remarkably (Figs. 3, 4).

## Discussion

In the present study, GLURP was used as pure antigen together with a crude antigen preparation containing many antigens. Generally, although the IgG antibody levels against the crude *P. falciparum* antigen were found to be significantly higher than those against GLURP, both have been found to correlate with protection against malaria [53, 54]. The concentrations of both antibodies showed a decline with IPTp uptake probably as a result of decay in antibodies or as a result of the clearance of parasitaemia by treatment with SP. This is consistent with other studies [43, 48, 55, 56], which also found a decline in the levels of antibodies against several antigens during pregnancy and after delivery. The decrease in antibody levels is believed to be linked with pregnancy-specific malaria immunity rather than to non-pregnancy associated malaria antigens [48] as speculated elsewhere [50] or possibly to short-lived responses in the absence of infection [43, 55].

HIV-infected pregnant women are known to also exhibit low levels of antibodies to *P. falciparum* antigens [57, 58] but this study did not collect information on HIV status of the study participants and therefore was unable to find any linkage for the low levels of IgG among the study participants. Again, previous malaria episodes have been demonstrated to be associated with increased antibody levels [59] but this was not explored in the present study. Another interesting factor associated with failure to acquire protective PAM immunity is exposure-reducing interventions [41]. Exposure to ITN interventions either early or later in pregnancy have also been shown to reduce malaria immunity but ITN usage among the study participants was very low (5%) as reported in an earlier study [50]. Analysis of the effect of the ITN use on the antibody levels could not be done due to the low numbers. Antibody levels could also be affected by seasonal change [60] or any particular pregnancy [61, 62] but there was a general decline among the pregnant women who were recruited at the beginning of the study and those recruited later as the recruitment period was short (July to August) and there was minimum or no variation in the season.

Although there were differences expected in parity of the women, both primigravidae and multigravidae showed a decline in antibody levels in support of the low prevalence rate. Another factor of interest is the plasma volume expansion associated with pregnancy, which might contribute to the overall decrease in antibody levels, attributed to low or lack of exposure [56]. A virtual anaemia is caused by an increase in circulating blood plasma. As a result, there are fewer red blood cells per ml of plasma compared to the blood of the non-pregnant, thereby making the blood of the pregnant woman more diluted and less concentrated in antibodies. Until recently this physiological phenomenon has been taken to mean that the pregnant woman is anaemic and therefore mean corpuscular volume (MCV) is a more accurate measure of anaemia. Nevertheless, a comparison with the above studies shows low anaemia rates for the women in the present study population [50]. This is supported by reports of a study in a rural district in Ghana with anaemia prevalence rate of 57.1% (haemoglobin (Hb) <10 g/dl) among pregnant women. The study concluded that anaemia was more prevalent among rural pregnant women than those in the urban areas of the same district (P = 0.01) [29].

The low prevalence of moderate and severe anaemia is supported by the finding of low prevalence of peripheral blood parasitaemia. Contrary to earlier studies, malaria among pregnant women living in areas of malaria has been reported to be symptomatic [5, 63]. Another study in an area of high and intense malaria transmission has shown the overall prevalence of malaria among women attending ANC to be about 47% with anaemia rate of 72% (Hb < 11 g/dl) and severe anaemia rate of 2% (Hb < 7 g/dl) [64]. This still underscores the fact that malaria remains the major cause of maternal anaemia in Madina and therefore programmes and policies aimed at the control of anaemia in pregnant women in Madina should focus primarily on malaria.

The peaks of IgM and IgG antibody levels post-IPT1, also around the second trimester, may indicate increased susceptibility to malaria, which usually occurs in the second trimester [28, 65]. The presence of IgM suggests that the women were still infected despite the IPTp they were receiving during pregnancy or that the parasites were possibly resistant to SP to some degree [66]. However, SP-IPTp has recently been shown to clear parasitaemia in asymptomatic women [67]. The steady decline in anti-IgM against both crude antigen and GLURP from baseline and after each dose of IPTp towards delivery may reflect the lower exposure to malaria, since infection and intra-erythrocytic replication of parasites is required for the induction of these antibodies. This shows that any methods which either reduce exposure to mosquito bites, such as bed nets or interrupts erythrocytic replication of the parasites, such as anti-malarials, can effectively reduce antibody levels against blood-stage antigens as shown by the present study. The decline in IgG levels shown in this study has also been reported [47] and may reflect the use of IPTp. However, since the threshold for protection of these antibodies against malaria were not established in the present study it is impossible to determine whether these women were rendered susceptible to malaria as a result of IPTp use.

The lower levels of anti-crude IgM antibodies in multigravidae as compared to primigravidae, reflects possible sub-microscopic parasitaemia carriage in primigravidae. This reflects new infections which could mean that most of the primigravidae might have experienced current infections compared to multigravidae, which is inconcert with the literature.

IgG against the crude antigen and GLURP were present in both placenta and cord blood even though the mean concentrations in cord blood were less than in the placenta, as found in other studies [68, 69]. A possible explanation is that in women with placental malaria, the anti-malarial antibodies bind to malaria antigen and become trapped in the placenta as immune complexes [70]. Further, the lower antibody levels in cord blood may be due to the thickening of the placental membranes as a result of malaria infection [71], a condition which may serve as a barrier to the efficient transfer of maternal proteins in a more generalized manner and which is similar to what happens with the transfer of low levels of anti-tetanus antibodies by women with placental malaria [72]. However, in the present study peripheral blood parasitaemia was only 5%, while placental malaria was present in samples examined (2.5%) [50]. Future studies should include other non-malarial antigens, including measles antigen, to provide better understanding of trans-placental transfer of maternal antibody.

There are presently no specific quantities of malaria antibodies that correlate with clinical protection [73] and therefore it is not possible to state whether or not the antibody concentrations in the cord blood were enough to offer protection to the infants born to study participants. Children with particularly high levels of antibodies at birth can remain antibody positive (in the absence of active immunization by infection) up to 11 months but in Ghana the median duration of maternal antibodies to a crude *P. falciparum* schizont extract was 14 weeks [74]. Maternal antibody falls to minimal levels by the 4th month of life while infant antibody titres began to rise from about 6 months of age, following active immunization by exposure [75]. Newborn infants in endemic areas are markedly resistant to *falciparum* malaria due to high levels of anti-malarial IgG passively transferred across the placenta. Consequently, severe disease is rare during the first few months of life, and infections tend to be low density and relatively asymptomatic during this period. However, congenital parasitaemia may present 2–3 months after delivery when maternal antibodies wear off [16].

There are conflicting views about the protection conferred on infants by maternal antibodies with the majority of the studies concluding that despite the small numbers in their respective studies, maternal antibodies do protect infants from clinical malaria and high parasitaemia irrespective of the presence or absence of maternally derived antibody, but do not protect infants from malaria infection, most of whom are asymptomatic [76]. There is also evidence for an association between decreasing levels of maternally derived, malaria-specific IgG and increasing risk of clinical malaria at a population level, but the numbers of children in most of the studies were not large enough to provide any convincing analysis [71]. The small numbers of children born to the study is a challenge. Women gave their consent to participate in the study and were recruited based upon their delivery at the hospital. However, socio-cultural practices made about 60% of them deliver outside the study-designated facility. They could not be replaced because the study was time bound. The study also provided a delivery package as incentive and participants received priority consultation at the ANC.

None of the children in the present study registered hyperpyrexia (body temperature >36.5 °C) during the post-natal follow-up to justify the collection of blood samples as per the approved protocol. There are many reasons attributed to protection in childhood. These range from the biting habits of anopheline mosquitoes that have preference to for blood of adults and older children, to protection by breast milk constituents. Others are the physiologic barrier by foetal haemoglobin (HbF), resulting in the delay of parasite development and protection from severe illness [32]. The introduction of IPTp has recently been shown to be associated with increased risk of severe malaria among infants [45]. This has serious implications for the use IPTp and raises questions about the passive transfer of antibodies across the placenta as well as the quality and quantities transferred.

## Conclusions

The study concludes that intermittent preventive treatment reduces immunoglobulin G levels among pregnant women on IPTp due to the low prevalence of parasitaemia. The children born to the mothers were protected for 6 months and showed no clinical symptoms after follow-up and thus IPTp allowed the transfer of antibodies across the placenta. Future studies should look into other non-malarial antigens to provide better understanding of antibody transfer.

## Abbreviations

AG: Antigen
ANC: Antenatal clinic
CSA: chondroitin sulfate–A
DOT: direct observed therapy
GLURP: glutamate-rich protei
IG: immunoglobulin
IPTp: intermittent preventive treatment in pregnancy
IVB: intervillous blood
LBW: low birth weight
MCV: mean corpuscular volume
OD: optical density
PAM: pregnancy-associated malaria
PBS: phosphate buffer saline
SP: Sulfadoxine–pyrimethamine
VSA: variant surface antigen
